# Two Commercially Available Blood-Stabilization Reagents Serve as Potent Inactivators of Coronaviruses

**DOI:** 10.3390/pathogens12091082

**Published:** 2023-08-25

**Authors:** Joseph J. O’Connor, Lynden Voth, Jeremiah Athmer, Nicholas M. George, Christopher M. Connelly, Anthony R. Fehr

**Affiliations:** 1 Department of Molecular Biosciences, University of Kansas, Lawrence, KS 66045, USA; 2 Streck LLC, 7002 S. 109th Street, La Vista, Omaha, NE 68128, USAcconnelly@streck.com (C.M.C.)

**Keywords:** coronavirus, SARS-CoV-2, blood collection reagents, RNA stabilization

## Abstract

The continued circulation of SARS-CoV-2 and the increasing frequency of coronavirus (CoV) outbreaks over the decades demonstrates the enduring threat that the CoV family poses. There remains a significant need to develop tools to monitor and prevent the spread of these viruses. We tested blood-stabilization reagents from two commercially available blood collection tubes (BCTs) for their ability to inactivate three different coronaviruses (MHV, OC-43, and SARS-CoV-2) and stabilize their RNA. Both Cell-Free DNA BCT^®^ (cfDNA) and Cyto-Chex^®^ BCT (CytoChex) reagents reduced infectious virus in the buffer to below the limit of detection within 18 h of treatment, with some conditions showing this effect in as little as 3 h. CytoChex had more potent activity than cfDNA as in all cases it more rapidly reduced the actively replicating virus to the limit of detection. Despite the rapid inactivation of the virus, both reagents effectively preserved viral RNA for 7 days. Finally, both reagents accelerated viral inactivation in blood compared to the control samples. These results indicate that cfDNA and CytoChex could be used to inactivate and preserve CoV RNA for detection and further testing.

## 1. Introduction

Since it emerged in late 2019, the COVID-19 pandemic has remained a significant public health challenge. Its causative agent, severe acute respiratory syndrome coronavirus (SARS-CoV-2), is a member of the family *Coronaviridae*, which are enveloped, positive-sense RNA viruses with unusually large genomes around 30 kb [1]. SARS-CoV-2 is the third coronavirus (CoV) of clinical significance to arise from zoonotic transmission in the last twenty years [2,3,4]. Additional human coronaviruses (hCoVs), like hCoV-OC43 and −229 E, also likely zoonotic in origin, are endemic to the human population and are thought to cause 15–30% of common cold cases and can cause severe disease in immunocompromised patients [5]. SARS-CoV-2 patients experience flu-like symptoms including headache, chills, fatigue, and a persistent cough. Severe infections may result in acute respiratory distress (ARDS), pneumonia, hypoxia, cardiovascular disease, inflammatory dysregulation, and death [1]. Worldwide, these infections have caused deaths in the millions and cases in the hundreds of millions [6]. The continued prevalence of SARS-CoV-2, even in areas with high rates of vaccination, demonstrates the need for diverse strategies for long-term case surveillance, mitigation strategies, and treatments.

Infection with SARS-CoV-2 can become systemic, targeting multiple organs such as the kidneys, liver, heart, brain, and blood [7], which can present as a variety of disease symptoms. The virus, likewise, has been detected in the organs of mice infected with a mouse-adapted SARS-CoV-2, including the heart, liver, kidney, spleen, and the intestines [8]. Notably, SARS-CoV-2 can infect primary cardiomyocytes and endothelial cells, and can cause a number of cardiovascular complications, including myocardial injury, myocarditis, pericarditis, cardiomyopathy, arrhythmia, heart failure, cardiac arrest, and others [9,10,11]. Considering the potential for SARS-CoV-2 to circulate in the blood, reagents that potently inactivate CoVs in the blood without destroying viral RNA could be valuable in the safe handling and testing of patient samples. Here, we investigated the efficacy of two commercially available blood-stabilization reagents, cfDNA and CytoChex, as viral inactivation buffers.

Previous data have demonstrated the utility of cfDNA in preserving the integrity of patient plasma cell-free DNA levels and methylation patterns in blood draw samples [12,13]. CytoChex has established uses in preserving cellular biomarkers for flow cytometry and has been shown to inactivate HIV in patient blood samples [14,15]. Using three members of the *Coronaviridae* family: hCoV-OC43, SARS-CoV-2, and mouse hepatitis virus (MHV-A59)—a model mouse coronavirus, we sought to determine whether these reagents would similarly inactivate CoVs while preserving relevant biomarkers. Room temperature incubation of all CoVs tested with either reagent resulted in near-complete reductions in viral infectivity after 18 h, with some conditions demonstrating inactivation in as little as 3 h. However, viral RNA was stable for up to 7 days. Finally, both reagents accelerated CoV inactivation in human blood compared to non-treated control samples. These results illustrate the applicability of these reagents to assist in the safe handling and testing of patient samples potentially infected with CoVs.

## 2. Materials and Methods

### 2.1. Cell Culture

HeLa cells expressing carcinoembryonic antigen-related cell adhesion molecule 1 (CEACAM1) as the MHV entry receptor (HeLa-MVR), 17Cl-1 mouse fibroblasts, HRT-18 human adenocarcinoma cells (ATCC), and Vero E6 green monkey kidney cell lines were grown in Dulbecco’s modified Eagle medium (DMEM) supplemented with 10% fetal bovine serum, 100 U/mL penicillin, 100 mg/mL streptomycin, HEPES, sodium pyruvate, nonessential amino acids, and L-glutamine. HeLa-MVR, 17Cl-1, and Vero E6 cell lines were generously provided by Stanley Perlman, University of Iowa.

### 2.2. MTT Viability Assay

Cells were seeded in 96-well plates at concentrations between 1 × 10^5^ and 1 × 10^6^ cells/mL. Monolayers were treated with various concentrations of reagent diluted in media. Cells were incubated in the presence of CytoChex or cfDNA at indicated dilutions for 20 h at 37 °C. Viability was assessed using CyQUANT™ MTT Cell Viability Assay (Life Technologies, Eugene, OR, USA) according to the manufacturer’s protocol.

### 2.3. Virus Inactivation

Amounts of 100 μL of incubation mixture, containing HBSS, between 2.8 × 10^5^ TCID_50_/_mL_ and 3.0 × 10^7^ PFU/mL, and either cfDNA or CytoChex reagents at 1:50 or 1:66 final dilutions, respectively, were incubated at room temperature for between 3 and 18 h. At each time point, mixtures were frozen at −80 °C before being diluted beyond the cytotoxicity threshold (the concentration of each respective reagent where the relative number of viable cells was comparable to mock-treated control cells, established via MTT assay). This was carried out to confirm whether cells (infected and control) were viable for infection/titer assay use. Diluted sample volumes were plated on the appropriate cell type. Viral titers in the form of TCID_50_/_mL_ (hCoV-OC43, SARS-CoV-2) or PFU/mL (MHV-A59) were assayed at 24 (MHV-A59), 48 (SARS-CoV-2), or 96 (hCoV-OC43) hours post-infection. These titration techniques were selected for each virus based on the distinguishability of plaque or cytopathic effect (CPE) and are considered for the purposes of this study to be interchangeable assays of viral replication. Limits of detection were defined as one half of the titer calculated from a single positive result (plaque or TCID_50_-well) at the lowest dilution.

### 2.4. Virus Inactivation in Blood

Two volumes of human blood, from two anonymous and presumed healthy individual donors, were procured from Streck with informed consent. Each volume of blood was spiked with 1% volume MHV-A59 stocks for final concentrations of 2.45 × 10^6^ PFU/mL. Aliquots of 1 mL of blood/virus mixture were added to tubes containing Streck Cell-Free DNA BCT^®^ (cfDNA) or Cyto-Chex^®^ BCT (CytoChex) reagents, with K_3_EDTA as the anticoagulant, using reagent-to-sample dilution factors of 1:50 for cfDNA, 1:66 for CytoChex, and 1:50 for HBSS as a mock control. Samples were incubated at room temperature for the indicated times. The plasma fraction was harvested in 1 mL volumes of the incubation mixture, centrifuged for 10 min at 3250× *g*, and snap frozen in a dry ice/methanol bath. Viral titers from the plasma fraction were determined by plaque assay on HeLa-MVRs.

### 2.5. RNA Stability Assay

MHV-A59 (1% final concentration from a 3 × 10^7^ PFU/mL stock) was incubated in duplicate at room temperature in the presence of HBSS, cfDNA (1:50 dilution), and CytoChex (1:66 dilution) for 0 to 7 days. Viral RNA was then isolated using the QIAamp Circulating Nucleic Acid Kit (Qiagen, Hilden, Germany) according to the manufacturer’s included miRNA protocol where the 60 °C incubation step was extended from thirty minutes to two hours to ensure complete yield recovery. Viral genomic RNA was converted to cDNA using 1 µg isolated RNA and the iScript^TM^ cDNA Synthesis Kit (Bio-Rad, Hercules, CA, USA). Viral genomic RNA levels were measured via quantitative PCR (qPCR) using PowerUp SYBR Green master mix (ThermoFisher, Waltham, MA, USA)) and the following primers that target nsp12: Forward primer 5′-AGGGAGTTTGACCTTGTTCAG-3′ and Reverse primer 5′-ATAATGCACCTGTCATCCTCG-3′.

### 2.6. Statistical Analysis

**Figure 1:**
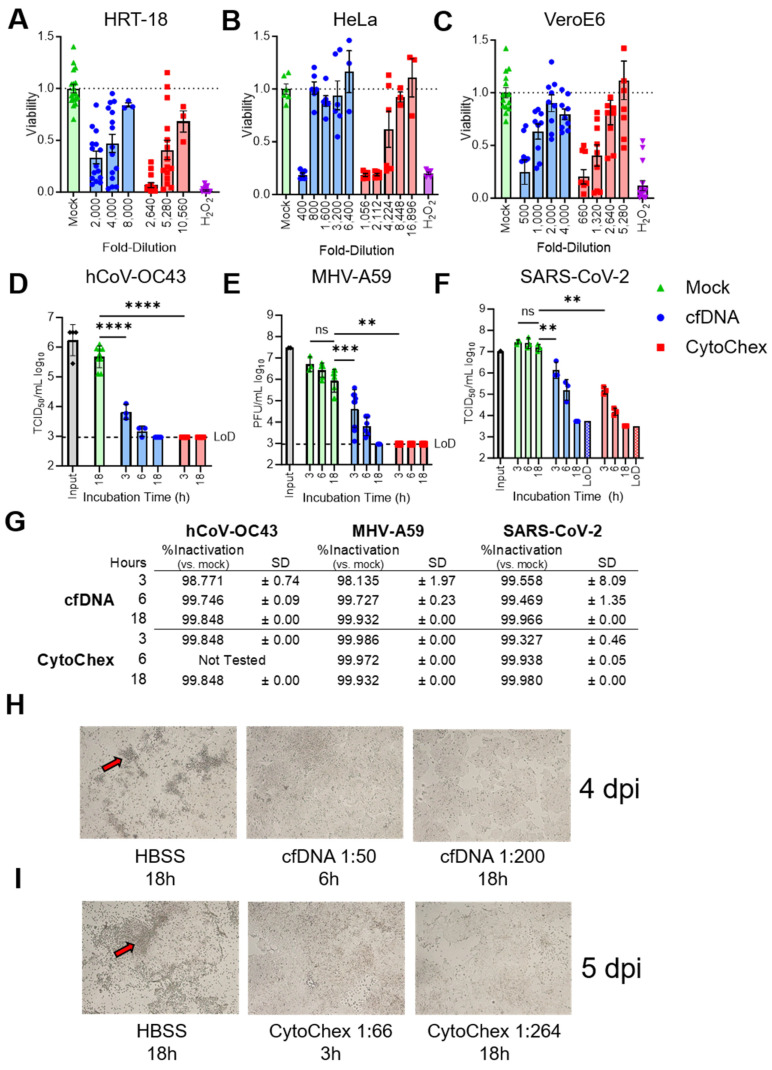
Blood collection reagents inactivate coronaviruses (CoVs). (**A**–**C**) Cells permissive for replication of each CoV were seeded in 96-well plates between 1 × 10^5^ and 1 × 10^6^ cells/mL. Cells were treated for 20 h with the indicated dilutions of cfDNA and CytoChex reagents. Viability of these cells was assayed in triplicate via CYQUANT™ MTT Cell Viability Assay. Mock-treated samples containing HBSS served as an untreated control. The relative viability was determined by normalizing the cfDNA or CytoChex reagent treated cells to HBSS treated cells. The number of replicates for each treatment are indicated above each data point. At least two separate experiments were performed for each virus. (**D**–**F**) Indicated CoVs were incubated with a 1:50 and a 1:66 dilution of cfDNA and CytoChex, respectively, for 3, 6, or 18 h at room temperature. The limit of detection (LoD) is indicated by a dashed line in (**D**,**E**), and as a separate data point in F and is based on the highest dilution used for TCID_50_. Significant comparisons were made between the 18 hr mock and 3 hr mock or treated samples in each graph. Significant *p* values are denoted with asterisks: **, *p* < 0.01; ***, *p* < 0.001; and ****, *p* < 0.0001. (**G**) % Inactivation of CoVs by cfDNA and CytoChex in assays described in (**D**–**F**). (**H**,**I**) OC43 at either 3.16 × 10^5^ (**H**) or 7.5 × 10^5^ TCID_50_/_mL_ (**I**) was incubated with cfDNA (**H**) or CytoChex (**I**) at room temperature for 3, 6, or 18 h. After incubation, reagent mixtures and mock controls were then diluted to concentrations that were not cytotoxic (cfDNA 1:20,000; CytoChex 1:26,400). The total volume of these dilutions was plated on multiple plates of HRT-18 cells. Plates were examined visually for CPE, signifying OC43 replication (red arrows) at 4 or 5 dpi. Images are from a single experiment representative of two independent trials for each reagent.

**Figure 2:**
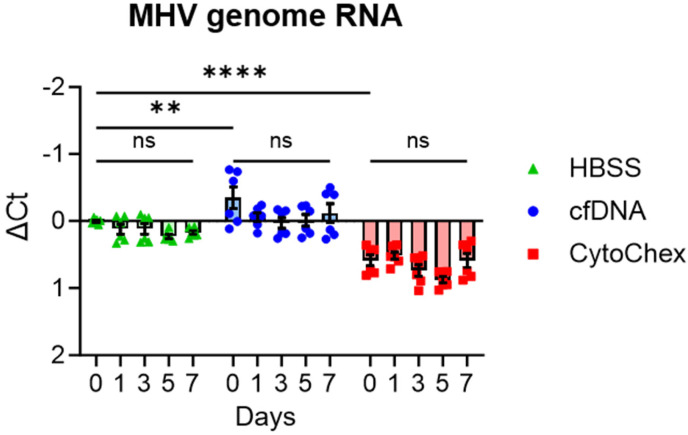
cfDNA and CytoChex have little to no effect on viral RNA levels over 7 days. MHV-A59 was incubated with indicated reagents and assayed for viral genome abundance via qPCR. MHV-A59 (1%) with a titer of 3 × 10^7^ PFU/mL was incubated with cfDNA (1:50) and CytoChex (1:66) at room temperature over the course of 7 days. Viral RNA was isolated using the QIAamp Circulating Nucleic Acid Kit as per the manufacturer’s protocol. Day 0 samples were incubated with the reagent for 30 min. Data represent combined values of two independent trials (*n* = 6). Significant *p* values are denoted with asterisks: **, *p* < 0.01; and ****, *p* < 0.0001.

All statistical analyses were carried out using two-way ANOVA with either Tukey’s (Figure 1D–F) or Dunnet’s (Figure 2) multiple comparisons test. Significant *p* values are denoted with asterisks: *, *p* < 0.05; **, *p* < 0.01; ***, *p* < 0.001; and ****, *p* < 0.0001. Graphs are expressed as geometric means ± geometric standard deviations (for viral titers) or standard errors of the means (SEM) (for qPCR). All data were analyzed using GraphPad Prism software v9.

## 3. Results and Discussion

### 3.1. Blood Stabilization Reagents Inactivate CoVs

Streck Cell-Free DNA BCT^®^ (cfDNA; Streck LLC, La Vista, NE U.S.A.) and Cyto-Chex^®^ (CytoChex; Streck LLC, La Vista, NE U.S.A.) are two commercially available blood collection tubes used for stabilizing whole blood samples for transport and analysis post-venipuncture. For the purpose of these studies, we tested each of the two proprietary blood-stabilization reagents, contained in the respective tubes, for their ability to inactivate CoVs. Each reagent is known to be toxic to cells, so we first determined the lowest dilution of each reagent at which we could reliably obtain viral titers (Figure 1A–C). For cfDNA, we tested three cell lines commonly used for CoV viral replication assays and found these dilutions to be 1:2000, 1:8000, and 1:800 on HRT18, HeLa-MVR, and Vero E6 cells, respectively. For CytoChex, these dilutions were 1:2640, 1:10,560, and 1:8448 on HRT18, Hela-MVR, and Vero E6 cells, respectively. Notably, viral titers could be reliably determined on cells with ≥50% viability as determined by an MTT viability assay. Next, we tested these reagents for their ability to inactivate OC43, MHV-A59, and SARS-CoV-2 in solution by measuring viral infectivity after incubation using either plaque assays of MHV-A59 on HeLa-MVR cells or tissue culture infectious dose assays (TCID_50_) of OC43 and SARS-CoV-2 on HRT-18 and VeroE6 cells, respectively (Figure 1D–F). The dilution of cfDNA (1:50) or CytoChex (1:66) reagents, representing the dilution of each reagent that occurs upon complete draw into a blood collection tube (the working concentration following blood draw as per the manufacturer’s instructions for use), were incubated with 2 × 10^6^ TCID_50_/_mL_ OC43, 2 × 10^7^ TCID_50_/_mL_ MHV-A59, or 1 × 10^7^ TCID_50_/_mL_ SARS-CoV-2 for 3, 6, or 18 h. By 18 h, each virus was reduced to the limit of detection (LoD) by each reagent, with % inactivation values ranging from 99.848% to 99.98% inactivation (Figure 1G). At 3 and 6 h after treatment, CytoChex more effectively inactivated virus in all cases, with % inactivation ranging between 99.327% and 99.972%, while cfDNA mediated inactivation ranged from 98.135% to 99.746%. All cfDNA and CytoChex samples treated for 3 h were highly significantly reduced compared to mock-treated samples at 18 h after treatment (Figure 1D–F).

To support the hypothesis that cfDNA and CytoChex led to the total inactivation of OC43, we diluted OC43 samples treated with cfDNA or CytoChex to a concentration that was not toxic to HRT-18 cells, then plated this entire volume of virus and reagent on HRT-18 cells. We then analyzed these cells over the course of 4–5 days for signs of CPE. No virus-induced CPE was identified from samples incubated with cfDNA or CytoChex, while CPE was easily identified throughout the plates in mock-treated samples at 4 (Figure 1H) and 5 (Figure 1I) days post-infection.

### 3.2. Blood Stabilization Reagents Have Limited to No Impact on Viral RNA Abundance

Having established that each of these reagents were capable of inactivating the infectious virus, we next sought to determine whether exposure to these reagents would cause viral RNA degradation within 7 days, a timeframe which is suitable for the potential downstream applications of these reagents. MHV-A59 was incubated with each reagent ranging from 30 min (day 0) to 7 days, after which RNA was isolated using the QIAamp Circulating Nucleic Acid Kitas per the manufacturer’s instructions. Treatment with HBSS alone had no impact on the abundance of viral RNA through 7 days. Similarly, cfDNA also had no impact on the abundance of viral RNA. Within 30 min, incubation with CytoChex increased C_t_ values by around one cycle, equating to an approximate twofold reduction in RNA; however, no further reduction in viral RNA levels was measured over the remaining 7 days (Figure 2). From these tests, we conclude that neither reagent causes substantial loss of viral RNA over 7 days, which indicates that these reagents would preserve viral RNA for detection in patient samples.

### 3.3. Blood Stabilization Reagents Accelerate Viral Inactivation in Human Blood

**Figure 3:**
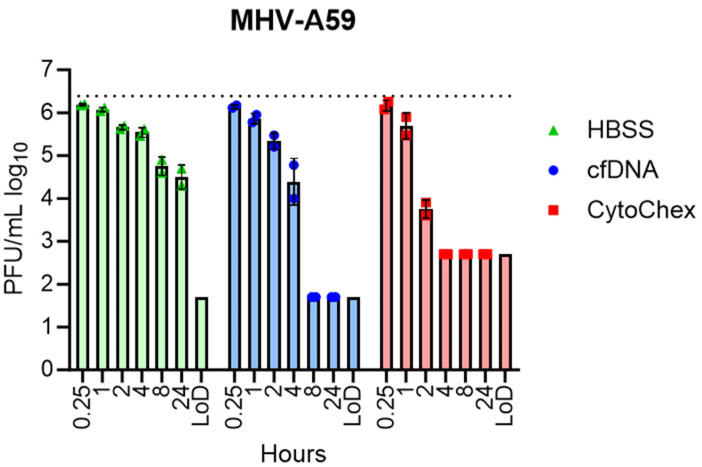
cfDNA and CytoChex reagents accelerate viral inactivation in human blood. Blood from a single donor was spiked with 1% MHV-A59 with a titer of 3 × 10^8^ PFU/mL. Spiked blood was added to blood collection reagents for final concentrations of 1:50 for cfDNA and 1:66 for CytoChex. Blood was incubated at room temperature for indicated times, then plasma fraction was harvested in 1 mL volumes and centrifuged for 10 min at 3250× *g* and snap-frozen in a dry ice/methanol bath. Viral titers were determined via plaque assay on HeLa-MVR cells. Data representative of two independent trials (*n* = 2).

Finally, as a proof of principle, each reagent was tested for its ability to inactivate CoVs in human blood. Due to the potential for donors to have SARS-CoV-2 or OC-43 antibodies in their blood, we utilized MHV-A59 to evaluate the ability of each reagent to inactivate CoVs in blood. Blood from two individual donors was spiked with MHV-A59 for a final titer of 2.45 × 10^6^ PFU/mL and added to tubes containing blood stabilization reagents, replicating the dilution of reagent in full blood draw samples or HBSS. All samples were then incubated at room temperature for between 15 min and 24 h, after which the plasma fraction was spin-harvested and snap-frozen in a dry ice/methanol bath. Blood treated with CytoChex and cfDNA consistently inactivated MHV-A59 more rapidly than did the HBSS control alone (Figure 3). CytoChex was more effective than cfDNA, inactivating MHV to the limit of detection within 4 h of treatment, while cfDNA required about double the incubation time, though it should be noted that CytoChex demonstrated higher cytotoxicity, increasing the limit of detection for these samples. These results demonstrate that both reagents accelerate the inactivation of infectious coronavirus in patient blood samples.

From these experiments, we conclude that the blood-stabilization reagents cfDNA and CytoChex may serve as potent inactivators of coronaviruses. These reagents, when in contact with viral particles, can render them inactive without reducing the presence of viral RNA. This study suggests that samples treated with these reagents pose a lower contact risk without eliminating the viral biomarkers necessary for additional detection of coronaviruses in blood or other samples. This information, coupled with previous work demonstrating a similar effect with HIV in patient samples [14,15], suggests the potential for an even broader applicability of these reagents to additional human pathogenic viruses.

## Data Availability

All primary data associated with these results are available upon request.
